# Physiological arousal explains infant gaze following in various social contexts

**DOI:** 10.1098/rsos.220592

**Published:** 2022-08-17

**Authors:** Mitsuhiko Ishikawa, Atsushi Senju, Masaharu Kato, Shoji Itakura

**Affiliations:** ^1^ Centre for Baby Science, Doshisha University, 4-1-1 Kizugawadai, Kizugawa, Kyoto 619-0295, Japan; ^2^ Centre for Brain and Cognitive Development, Birkbeck College, University of London, Malet Street, London WC1E 7HX, UK; ^3^ Research Centre for Child Mental Development, Hamamatsu University School of Medicine, 1-20-1 Handayama, Higashi-ku, Hamamatsu, Sizuoka 431-3192, Japan

**Keywords:** eye contact, reliability, gaze following, heart rate, social decision-making

## Abstract

Gaze following (GF) is fundamental to central aspects of human sociocognitive development, such as acquiring language and cultural learning. Studies have shown that infant GF is not a simple reflexive orientation to an adult's eye movement. By contrast, infants adaptively modulate GF behaviour depending on the social context. However, arguably, the neurophysiological mechanisms underlying contextual modulation of GF remain somewhat unexplored. In this study, we tested the proposition about whether the contextual modulation of infant GF is mediated by the infant's heart rate (HR), which indicates the infant's physiological arousal. Forty-one 6- to 9-month-old infants participated in this study, and infants observed either a reliable face, which looked towards the location of an object, or an unreliable face, which looked away from the location of an object. Thereafter, the infants watched a video of the same model making eye contact or not making any ostensive signals, before shifting their gaze towards one of the two objects. We revealed that reliability and eye contact acted independently to increase HR, which then fully mediates the effects of these social cues on the frequency of GF. Results suggest that each social cue independently enhances physiological arousal, which then accumulatively predicts the likelihood of infant GF behaviour.

## 1. Introduction

A hallmark of human intelligence is the exceptional capacity to learn from other humans [1,2]. From very nearly the earliest stage of life, learning from other conspecifics is essential for human infants to accrue information relevant to their survival and acculturation, including the acquisition of generalizable knowledge or usable skills from another individual [3]. Especially in preverbal infants, adults' gaze direction plays a critical role in the process of social learning [4]. Therefore, it is not surprising that infants’ gaze-following (GF) behaviour has become a core topic in the cognitive, developmental, social, and comparative psychologies and neurosciences [5].

Early studies conceptualized infant GF as an automatic or reflexive shift of infant visual attention, prompted by adults' gaze direction [6]. However, studies over the past decade have begun to suggest that infant GF is context-dependent, in which the various social contexts modulate the probability of infant GF. Here, we present two examples of such contexts—ostensive signals and the reliability of a communicative partner.

First, infants follow others’ gaze when accompanied by ostensive signals: the signals that are hypothesized to convey an adult's communicative intent [7,8]. Senju & Csibra [7] showed that 6.5-month-old infants follow others' gaze when it followed eye contact or infant-directed speech (ostensive signals), but not when it followed non-ostensive but eye-catching stimuli (e.g. non-social animation overlaid on the actor's face). Csibra & Gergely [3] interpreted this (and convergent) as evidence that infants follow others' gaze when they referred to the topic of communication within the framework of ostensive-referential communication and suggested the theory of natural pedagogy.

Second, the trait of the communicative partner, namely, reliability as an informant, also modulates infant GF and subsequent selective learning [9,10]. For example, it has been reported that 8-month-old infants can track the reliability of potential informants and use this information selectively to modify their future behaviour [11]. It has been shown that infants modulate their social behaviour such as imitation [12,13] and GF [14] depending on others’ reliability. In two of these studies [12,14], infants first observed an experimenter looking inside a container that either contained a toy (the reliable looker condition) or was empty (the unreliable looker condition). In this task, infants could expect either to find a toy when the experimenter gazed inside a container or to find the container empty. After the observation, infants engaged in the GF task [14]; thus, it was possible to test whether infants' GF would be modulated by their knowledge of the experimenter's gaze reliability. This kind of observing the experience of another's gaze behaviour made it possible for the infants to evaluate the other's reliability as an informant, and the infants thereby accordingly modulated their subsequent social engagement.

The studies discussed above demonstrated that the presence of eye contact and the reliability of the communicative partner facilitate infants' GF [7,14]. However, we should note that these two contextual factors are qualitatively different: the presence of eye contact is an immediate visual context, while the factor of reliability is knowledge acquired from prior experiences requiring memory modulation. However, because both factors modulate infants’ GF, these different contextual modulations of GF may share common cognitive substrates before emerging as GF behaviour. We hypothesize that infants calculate ‘action value’, or a value assigned to each action alternative (e.g. to follow or not to follow gaze) in a given social context (e.g. when the communicative partner is making eye contact). In this framework of value calculation, infants are expected to select that action (e.g. to follow the gaze) which has a higher value (e.g. leads to a learning opportunity or a social affiliation) than alternative actions (e.g. not to follow the gaze and to look at another object in the scene instead). The framework also assumes that infants expect a reward (e.g. informational values such as a name of an unfamiliar object or social values such as a positive response from the communicating partner) after executing the action, which is again based on the value calculations. From this perspective of an action value calculation, different contextual cues (e.g. ostensive signals and the reliability of a communicative partner) are processed and integrated as a value assigned to each action alternative, which will then determine the infant's decision to execute the action alternative with the highest value.

Note that we are not the first to claim that infants' GF is value-driven action. For example, Triesch *et al*. [15] applied a reinforcement learning framework to computational modelling of the emergence of GF and suggested that infants’ GF is reinforced by rewarding experiences (looking at interesting things) in mother–infant GF situations, and infants select GF according to a given high action value. In addition, Deák *et al*. [16] argued that salient and strong reward signals might enhance the frequency of GF behaviour. Recently, Michel *et al*. [17] showed that screen-based training, in which they presented an animation as a reward when infants followed a person's gaze direction, could reinforce GF behaviour in 4-month-olds. The results suggest that infants' GF can be mediated by the reward expectation formed during reinforcement learning.

The brain's limbic system, especially the striatum and amygdala elements, is known as a core part of the reward system and is expected to play a major role in the action value calculation. For example, previous studies using an fMRI technique have shown that the striatum is responsible for reward anticipation and prediction error [18,19] and the amygdala contributes to assigning a value to social stimuli [20]. Critically, these core regions of the reward system are anatomically close to the insula, the anterior cingulate cortex (ACC), and the hypothalamus, [21], which play a critical role in modulating sympathetic activities. Hence, it is expected that reward processing in the limbic system can modulate physiological states [22–24].

Several lines of evidence support the claim that reward processing correlates with the modulation of physiological states. First, studies using the simultaneous measurement of brain activity and physiological states during decision-making have reported that the brain activation network in the reward system, the insula, and the ACC correlates with the increase in physiological states [25,26]. Second, studies that used physiological measurements, such as heart rate (HR), eye blink, pupil dilation and skin conductance responses, observed sympathetic excitation during reward expectations [27–29]. Similarly, previous studies have shown that increases in the HR codify anticipation of rewards in monetary incentive paradigms [30,31]. Finally, it has been reported that the HR initially accelerates in response to the relevant monetary reward cues, and it was suggested that the HR accelerates abruptly during mobilization for a rapid response [27]. It has been suggested that sympathetic excitation to reward-relevant cues may be induced in preparation for executing the necessary actions to obtain said rewards [32].

The current study also adopts the same assumption, that physiological arousal correlates with reward processing and value calculation. The study is built on recent studies from our laboratory, which used the change in HR as an index of physiological arousal during the GF situation. The study showed that eye contact elevated HR in infants and that HR increase predicted later GF [33]. In other words, it was suggested that an increase in physiological arousal mediates subsequent GF behaviour in such an experimental context.

However, the results we introduced above do not fully support our hypothesis that physiological arousal indicates a calculated action value, which integrates multiple socially relevant (or reward-predictive) cues. It could also mean that physiological arousal mediates the influence of a specific type of social context, the presence of an ostensive signal, on infant GF. To test this hypothesis further, it is essential to show that the increase in physiological arousal mediates the heightened probability of infant GF in other social contexts, independent of the presence or absence of ostensive signals. In other words, it is necessary to measure physiological states in GF situations with other contextual cues that would, likewise, be hypothesized to induce reward expectations and demonstrate that such other contextual cues would increase infant HR, which then mediates the increase in infant GF behaviour.

The current study is designed to test the prediction based on the hypothesis we summarized above, namely, that infants integrate multiple social cues based on an action value calculation, which is indicated by infant physiological arousal and explains subsequent infant GF behaviour. More specifically, the current study examined how an infant's HR changes in two separate social contexts, which have enhanced infant GF, the presence of ostensive signals (communication-predictive cues) and the looker's reliability (a learnt predictiveness or learnt value of others [10]). To manipulate a looker's reliability as an informant, we first showed gaze-cueing situations in which a female looked directly towards an object or away from it. Infants could thereby learn that they can acquire rewards by following the female gaze. Then, the same female appeared in a GF task with or without eye contact. Replicating the previous study, [33] we predicted that infants would show GF after eye contact, which would be mediated by an increased HR. In addition, we predicted that if the looker gazed towards an object in the gaze-cueing situation (a reliable informant), infants would show a more frequent GF towards the same looker, which is also mediated by an increased HR. If infants' GF behaviour is determined by the reward expectation indexed by HR, which we hypothesize as an index of action value calculations in a certain social context, these two signals independently modulate infants’ HR, which would then fully mediate the effects of both social cues (reliability and eye contact) on GF. We also explored whether the effects of reliability and eye contact on GF were additive (i.e. two factors summate to predict the probability of GF) or disjunctive (i.e. the presence of either signal is sufficient to maximize GF, there being no additive effect found in terms of the other signal).

## 2. Methods

### 2.1. Participants

The final sample for analysis consisted of forty-one 6- to 9-month-old infants who completed the study (mean age, 237 days old; range, 180–295 days old) with 21 infants in the reliable looker condition (11 female and 10 male) and 20 in the unreliable looker condition (8 female and 12 male). Previous studies have reported that infants from six months old showed GF in screen-based experimental settings [7,34], and that there was no difference in the GF frequency between six- and nine-month-old infants [35]. The sample size was determined based on a study examining the effects of a looker's reliability on infant GF [14]. Using the effect size from the previous study (*f* = 0.71), we conducted an *a priori* power analysis with G*Power [36]. The results indicated that with 20 participants per group, we would have achieved above 95% power, with alpha at 0.05, to find the effects of reliability on infants' GF. The estimated sample size was also sufficient to determine the effects of eye contact on infants’ HR.

### 2.2. Apparatus

We used a Tobii Spectrum Eye Tracker (Tobii pro Lab 1.118, Tobii Technology, Stockholm, Sweden) to record eye movements during the presentation of the stimuli. The sampling rate was 120 Hz. Participants were seated approximately 60 cm from the monitor, on the carer's lap. Before recording the infants' eye movements, a nine-point calibration was conducted.

For the HR recording, we used a BIOPAC MP160 (BIOPAC Systems, CA, USA) and BioNomadix (BIOPAC Systems, CA, USA) with a 3-lead ECG to measure the ECG data at a 1000 Hz sampling rate. Before attaching the electrodes, we used rubbing alcohol to reduce impedance.

### 2.3. Stimuli and procedure

**Figure 1.**
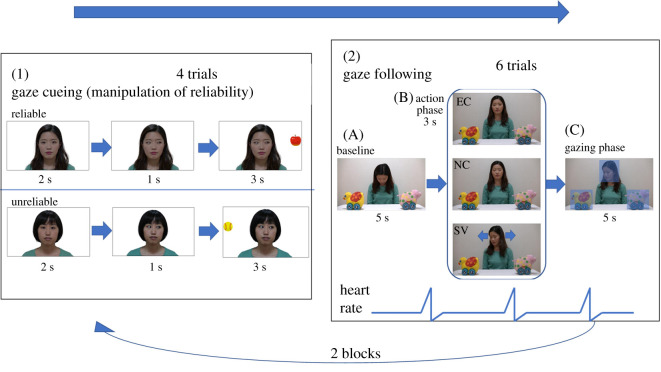
Structure of the experiment. (1) Illustration of the gaze-cueing situations. In the reliable group, an object appeared consistent with the looker's gaze direction, while another object appeared on the opposite side of the looker's gaze direction. (2) Selected frames of the stimulus videos in the GF task, including AOIs for analysis. All videos started with the baseline phase (A), followed by the action phase (B) and the gazing phase (C). The action phase consisted of three conditions: eye contact (EC), no cue (NC) and shivering (SV). Models represented in the figure have provided written permission to publish the images in all formats.

The infants participated in two blocks of tasks. Each block consisted of observation of four trials of gaze-cueing situations (i.e. induction of one of the reliability conditions), followed by six trials of the GF task. This sequence of a block was repeated twice for each participant, which resulted in each infant observing eight trials of the gaze-cueing situations (4 trials × 2 blocks) and performing 12 trials in the GF task (6 trials × 2 blocks). Both blocks were identical per participant. Figure 1 shows examples of the stimuli used during the gaze-cueing situations and the GF task. Completing all the trials takes about seven minutes.

### 2.4. Manipulation of the reliability of the actor

To manipulate the looker's social characteristics as an informant, we had infants observe gaze-cueing situations before the GF task (figure 1(1)). We modified the procedures and stimuli used by Ishikawa *et al*. [37]. There were three types of faces: (1) a direct gaze face for a pre-cueing stimulus, (2) a right-gazing face, and (3) a left-gazing face. A target object was placed approximately 15° to the left or right of the centre of the screen per trial. We used four female faces and six colourful illustrations as stimuli. The infants viewed four trials for each block. Each trial began with a picture of a female looking directly towards the infant (2 s), and after that, the female shifted her gaze direction either right or left (1 s), with no objects in either direction. Finally, an object appeared in a location being looked at by the actor (reliable looker) or in a location on the opposite side of the actor's face (unreliable looker) for 3 s. Two reliability conditions were allocated as a between-subjects factor, whereby half the infants only observed a reliable model and the other half only observed an unreliable model. Target objects were chosen randomly from six colourful pictures, such as an apple and a ball. The direction of the model's gaze was counterbalanced in ABBA order. Half of the infants saw a leftward gaze in the first trial, and the other half saw a rightward gaze first. In a block, the GF task was continuously started after four trial observations of gaze-cueing situations.

### 2.5. Gaze-following task

For the GF task, we applied the stimuli and procedures as used by Ishikawa & Itakura [33]. The infants watched a video of the same model who appeared in a gaze-cueing situation making eye contact or not displaying any ostensive signals, before shifting her gaze towards one of two objects. Each video clip begins with a female gazing downwards, seated at a table. Two toys that were not presented in the gaze-cueing situation were placed one on each side of the model (figure 1(2)). One of these two toys served as a target object, and the other toy served as a distractor object. The assignment of each toy as either a target or a distractor alternated between the trials. The stimulus videos for each trial consisted of three phases. At the baseline (figure 1(2)A), the model kept still for two seconds and gradually looked up with both eyes closed. Once the model was facing the front, the action phase was started (figure 1(2)B), which was different between conditions. In the eye contact (EC) condition, the model opened her eyes and maintained her direct gaze for three seconds. In the no cue (NC) condition, the model kept her eyes closed for three seconds. In the shivering (SV) condition, the model shook her head from side to side a few times for three seconds while keeping her eyes closed. The third phase was the gazing phase (figure 1(2)C). In this phase, the model directed her head at about 45° towards the target object and gazed at it for 5 seconds. The model opened her eyes just before turning her head in the NC and SV conditions. The model maintained a neutral facial expression and made no sound throughout the video clip.

The three communicative cue conditions were designed to run within-subject. Within each block, six trials were presented to each infant. The order of trials was quasi-randomized across the conditions. The assignment of the target object was randomized in each block. The model's gaze direction was counterbalanced in ABBABA order. Half of the infants watched a leftward gaze in the first trial, and the other half saw a rightward gaze first. The infant's attention was drawn to the centre of the screen by a Tobii attention getter before the start of each trial, on which the model's face appeared in the video clip. As mentioned earlier, the block was repeated twice, amounting to 12 trials per infant.

### 2.6. Data analysis

The sample's average percentage of gaze position data throughout the whole recording was 68.73% (s.d. = 12.95%, range: 45–96%). A Clearview fixation filter (Tobii Technology, Sweden) was applied for the eye-tracking data, one of the basic algorithms used in the Tobii eye-tracking software and has been used in previous infant studies [33]. As in the previous study [3], fixation was defined as a gaze recorded within a 50-pixel diameter for a minimum of 200 ms, and this criterion was applied to the raw eye-tracking data to determine the duration of any fixation.

### 2.7. Eye-tracking data

The measurement of GF was calculated based on whether the infant's first fixation towards an object preceded immediately by the fixation on the face area of interest (AOI) during the gazing phase (i.e. after the head turn started) went to the AOI of the object looked at by the model or towards the object opposite them. If the infant fixated on an object from the start of the gazing phase, it was not counted as constituting a looking behaviour, i.e. neither following nor not following. For example, if the infant fixated thus: (fixation 1) distractor, (fixation 2) distractor, (fixation 3) head, (fixation 4) target, the sequence of fixations 3 and 4 was counted as GF. We applied the same criteria that were used in the previous eye-tracking studies of infants' GF [33,38], and the likelihood of GF is 50% given this definition of GF. The face AOI includes the top of the head to the chin (figure 1). The infant's looking behaviour was coded as GF if the infant was fixated on the same object that the model gazed at. To be included in the analyses, it was required that infants elicited transition of fixation from the head towards an object during the gazing phase in at least three trials. Four infants who had fewer than three trials in looking at one of the two objects were excluded from the analysis.

Trials that excluded any fixations from the head towards an object during the gazing phase were likewise excluded from the analysis, and in total, 11 trials were excluded from the analysis of GF (EC: 3 trials, NC: 5 trials, SV: 3 trials).

We also analysed the total duration of fixations on the model's face for the baseline, the action, and the gazing phases separately.

### 2.8. Heart rate

We pre-processed the ECG signal using band-pass filtering with 0.5 and 40 Hz cut-off frequencies. Trials with excessive movement of the infant were excluded from the analysis before the calculation of the R-wave-to-R-wave (R-R) intervals. Eleven trials were excluded from the analysis of HR. All the excluded trials overlapped with the exclusion of eye-tracking data. In these excluded trials, infants were inattentive and/or fussy and thus, the moving gaze positions and ECG signals could not be measured properly. R peaks were detected by the detection algorithm of AcqKnowledge 3.9.0 software (BIOPAC Systems Inc., Santa Barbara, CA). Then, R-R intervals were checked visually to find missed beats, which were interpolated with the neighbouring R-R intervals. For the interpolation, we added the average durations of the neighbouring R-R intervals to the missed beats [39]. The ECG data were then separated into each of the three phases (baseline, action and gazing) and the average R-R intervals were calculated for each phase. Beats per minute were calculated for the analysis of HR. The change in average HRs, from the baseline to the action phase, for each trial, was calculated to examine whether HR increase predicts GF.

## 3. Results

Datasets used for the analysis are available in the electronic supplementary material.

### 3.1. Gaze following

For the analysis of GF, we conducted a generalized linear model (GLM) analysis with GF behaviour as the dependent variable, and the reliability of looker (reliable, unreliable) and the communicative cue condition (EC, NC, SV) as independent variables. The results showed a significant main effect of the communicative cue condition (estimate ± s.e. = 1.075 ± 0.3264, *p* = 0.0243). We used the Bonferroni correction for all *post hoc* tests. The EC condition showed a higher GF rate than the NC and SV conditions (EC versus NC: *p* = 0.017; EC versus SV: *p* = 0.022).

In addition, there was a main effect of reliability (estimate ± s.e. = 0.573 ± 0.3195, *p* = 0.0214), and the reliable group showed a more frequent GF than the unreliable group. There was no interaction effect between the communicative cue and reliability (estimate ± s.e. = 0.3684 ± 0.4624, *p* = 0.259).

**Figure 2.**
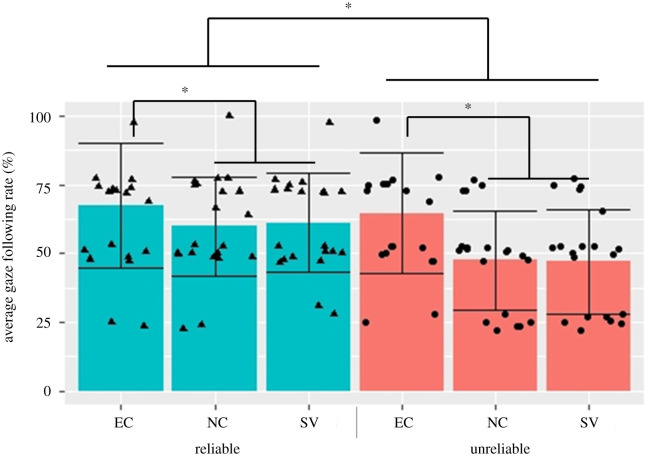
Results of gaze following during the gazing phase and the proportion of gaze following in each condition. The *x*-axis depicts the conditions and the *y*-axis depicts the percentage of gaze following.

Additionally, to examine whether the communicative cue and reliability facilitate GF, we conducted two-tailed *t*-tests between the GF rates and a chance level of 50% (figure 2). In the reliable group, infants showed significant GF in each communicative cue condition (EC: *M* = 67.43%, *t*_20_ = 3.313, *p* = 0.004, *d* = 0.74; NC: *M* = 59.86%, *t*_20_ = 2.225, *p* = 0.038, *d* = 0.49; SV: *M* = 61.1%, *t*_20_ = 2.564, *p* = 0.019, *d* = 0.57). By contrast, in the unreliable group, infants exhibited significant GF behaviour only in the EC condition (EC: *M* = 64.55%, *t*_19_ = 2.966, *p* = 0.008, *d* = 0.66; NC: *M* = 47.5%, *t*_19_ = −0.623, *p* = 0.541, *d* = −0.139; SV: *M* = 47.05%, *t*_19_ = −0.689, *p* = 0.499, *d* = −0.154).

### 3.2. Heart rate

**Figure 3.**
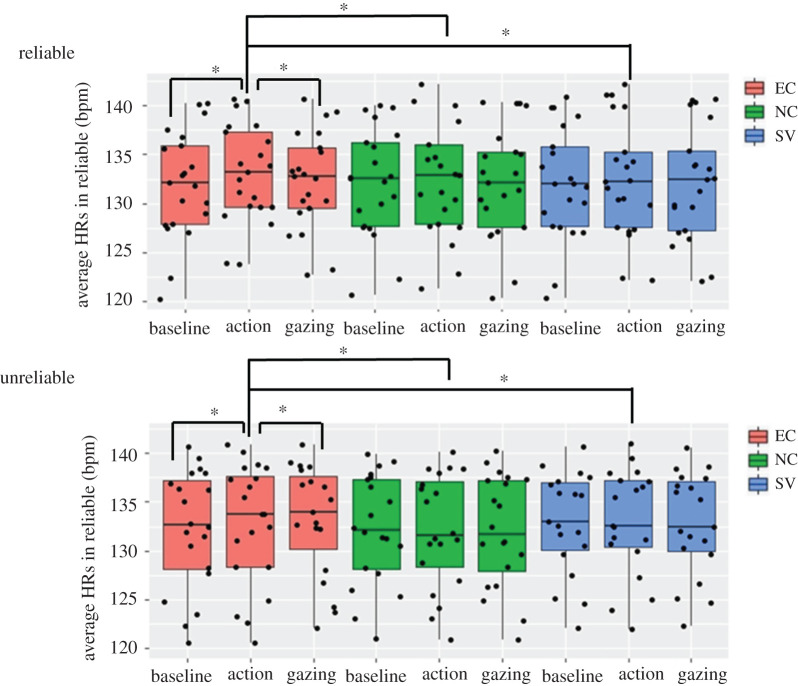
Mean HR levels during each phase for each condition. The *x*-axis depicts the video phase and the *y*-axis depicts the HR in beats per minute.

To examine how the infants' HR changed during the GF situations, a 2 × 3 × 3 ANOVA with two levels of reliability (reliable, unreliable), three levels of condition (EC, NC, SV), and three phase levels (baseline, action, gazing) was conducted (figure 3).

The results showed a significant interaction effect between the communicative cue and phase (*F*_4,156_ = 4.535, *p* = 0.002, $\eta_{p}^{2}=0.104$). All *post hoc* *t*-tests were corrected by the Bonferroni correction for multiple comparisons. The *post hoc* tests showed that an HR increase from the baseline (*M* = 132.11 bpm) to the action phase (*M* = 132.89 bpm, *p* < 0.001) and an HR decrease from the action phase to the gazing phase (*M* = 132.42 bpm, *p* = 0.001) were found in the EC condition. The SV condition showed a decrease in the HR from the action phase (*M* = 132.37 bpm) to the gazing phase (*M* = 132.09 bpm, *p* = 0.006). In the action phase, the average HR was higher for the EC condition than it was for the NC and SV conditions (EC versus NC: *p* = 0.032; EC versus SV: *p* = 0.039).

Additionally, a significant interaction effect between reliability and phase was found (*F*_2,78_ = 4.918, *p* = 0.01, $\eta_{p}^{2}=0.112$). The reliable group showed an increased HR from the baseline (*M* = 132.01 bpm) to the action phase (*M* = 132.68 bpm, *p* < 0.001) and a decreased HR from the action phase to the gazing phase (*M* = 132.16 bpm, *p* < 0.001). There was no interaction effect across communicative cue, reliability and phase (*F*_4,156_ = 0.062, *p* = 0.993, $\eta_{p}^{2}=0.002$).

### 3.3. Predicting gaze-following heart-rate increase

The behavioural results showed that reliability and the communicative cue condition enhanced the GF rate proportionately. In addition, results of HR showed that reliability and the communicative cue condition enhanced average HR levels proportionately. To examine how contextual factors (reliability and the communicative cue condition) modulate HR levels and induce GF, we performed GLM logistic regression analyses to predict GF by three factors—reliability, the communicative cue condition and HR increase levels (the degree of HR increase from the baseline to the action phase). The logistic regression of GLM enables us to investigate the influence of factors on the binary response (i.e. following or not) [40]; thus, it is possible to test whether the HR increase predicts the emergence of GF in each trial. The results of GLM analysis revealed that the HR increase rate predicted later GF (estimate ± s.e. = 76.45 ± 11.69, *Z* = 6.54, *p* < 0.001) in all conditions, with a higher HR increase pointing to GF behaviour. We also tested the difference between slopes predicting GF by HR increases across the two levels of reliability (reliable, unreliable) and three levels of condition (EC, NC, SV). Results indicated no significant differences between any levels of reliability or condition; in other words, the infants would show GF with the same frequency when they have the same levels of HR increase from the baseline to the action phase in all levels of reliability and condition.

### 3.4. Mediation of contextual modulation on gaze following by the heart-rate increase

**Figure 4.**
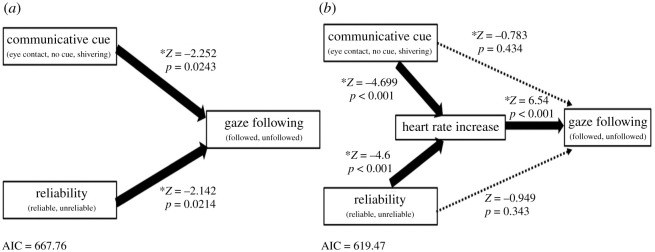
(*a*) Regression model predicting gaze following by communicative cue conditions (EC, NC, shivering) and reliability (reliable, unreliable), and (*b*) the mediation model shows that the HR increase mediates the relationship between communicative cue conditions and reliability and gaze following.

To examine whether the HR increase mediates the contextual modulation of GF, we compared model fitting between the regression model (figure 4*a*, model A) and the mediation model (figure 4*b*, model B).

The previous GLM analysis showed that the HR increase levels predict the emergence of GF. To examine whether HR increase levels mediate the effects of contextual factors on GF, we compared two models with and without HR levels predicting GF. As shown in the analysis of GF, the communicative cue condition and reliability predicted GF proportionately (communicative cue: estimate ± s.e. = 1.075 ± 0.3264, *Z* = −2.252, *p* = 0.0243; reliability: estimate ± s.e. = 0.573 ± 0.3195, *Z* = −2.142, *p* = 0.0214) without the factor of HR increase levels. Both the communicative cue condition and reliability predicted HR increase levels (communicative cue: estimate ± s.e. = 0.0111 ± 0.0049, *Z* = −4.699, *p* < 0.001; reliability: estimate ± s.e. = 0.0085 ± 0.0039, *Z* = −4.6, *p* < 0.001). However, with HR increase levels, the communicative cue condition and reliability did not show direct effects on GF (communicative cue: estimate ± s.e. = 0.1437 ± 0.1908, *Z* = −0.783, *p* = 0.434; reliability: estimate ± s.e. = 0.1565 ± 0.1864, *Z* = −0.949, *p* = 0.343). In other words, the increase in HR mediated between the communicative cue condition or reliability and GF. Model B (619.47) showed a smaller Akaike information criterion (AIC) than model A (667.76); thus, the mediation model was chosen as the better model.

### 3.5. Additional analysis

#### 3.5.1. Attention to the face area

To confirm that the infants learnt the actor's face equally in gaze-cueing situations, we compared the total duration of fixation on the face between reliable and unreliable using a *t*-test. There was no difference in the total duration of fixation on the face area between reliable (*M* = 1.23 s) and unreliable (*M* = 1.20 s). Infants looked at the face equally before the GF task.

In the GF task, because attention to the model's face may have affected infant HR and GF, we examined the infants' attention to the model's face across conditions in all three phases. We conducted an ANOVA using the duration of gaze at the model's face as the dependent variable across conditions for each phase (two levels of reliability: reliable, unreliable; three levels of condition: EC, NC, SV; three phase levels: baseline, action and gazing). There were no differences between any conditions or phases.

#### 3.5.2. Learning effects over the trials

Infants were engaged in two blocks of the experimental procedure; therefore, repeated trials might have affected infants' learning during the experiment. To examine potential learning effects over the trials, we compared GF frequency between the first block and the second block of the experiment across all conditions (two levels of reliability: reliable, unreliable; and the three levels of condition: EC, NC, SV) using a *χ*^2^-test. There was no significant difference between blocks ($\chi_{5}{\;}^{2}=4.645$, *p* = 0.461); thus, we concluded that there were no learning effects of repeated trials.

## 4. Discussion

The study revealed that the EC and reliability of the communicative partner modulate the frequency of infant GF and infant HR, respectively, suggesting that these two contextual effects on GF are independent of each other. While each contextual factor affected infant GF independently, the result of the mediation analysis showed that the increase in HR fully mediated the effects of both EC and reliability on GF. These results are in line with our hypothesis that the various social contexts are integrated as an action value, and the reward expectation is based on the calculated action value and indexed by the HR [27,32], which then modulates infant GF.

We replicated the finding that EC or reliability facilitates GF and showed that these facilitative effects are additive, with no significant interaction between these two factors. The results suggest that the pathways for the encoding of ostension and reliability are independent of each other, and the calculation of action value would only be integrated when the outputs of the pathways summatively modulate physiological arousal, which we hypothesized to represent an action value or a reward expectation based on the value calculation.

The results are consistent with our hypothesis that the physiological arousal before GF behaviour is an index of reward expectations because of action value calculation. Concurrently, previous adult studies have indicated that reward expectation induces high physiological arousal, indexed by skin conductance response and pupil dilation [29,41]. In addition, it has been shown that reward-predictive cues can enhance physiological arousal in infants and that pupil dilation in response to reward-associated cues predicts the success of associative learning [28]. Note that these studies used different measurements for physiological arousal, but as we discussed earlier in the Introduction, these different measurements all correlate with sympathetic excitation [27–29], which we hypothesized to relate to reward processing. With the current study, these other reports suggest that the ostensive signals, which have been described as cues that convey communicative intent to the social partner [3], would have activated reward systems as other communication acts, such as engaging with speech [42] and looking at the same object with the other person (joint attention) [43].

Based on the previous studies [27,32], we argue that the increase in HR represents a reward expectation in the current experimental context. However, we did not manipulate the levels of HRs and inferred the mechanisms of GF based on statistical modelling. We acknowledge that further evidence will be required to make a stronger claim about the relationship between physiological arousal, particularly those that correlate with the HR and possibly with those indexed by different measurements, and the calculations of action value. For example, high physiological arousal could also relate to high sensitivity and responsiveness to the external environment [44], a process that might not involve an action value calculation. To further examine whether the contextual modulation of infants’ HR (which then mediated GF) is based on the expectation of reward, future studies will need to examine whether infants' HR is correlated with activation in the brain's reward system and HR can index reward expectations in general. Future studies should also use a wider range of physiological measurements. For example, it has been reported that infants show pupil dilation to reward-predictive cues after they learnt the associations between cues and rewards [28], which makes pupil dilation another candidate physiological measurement. Note that this would require modification of the experimental paradigm because pupil diameters are affected by movements of dynamic stimuli regardless of cognitive processing [45], which would be problematic in the current experimental paradigm using dynamic video stimuli. Furthermore, it would be ideal for future studies to find a way to manipulate the physiological arousal of infants directly within the experimental setting and to examine the causal or mechanistic role of physiological arousal in infant GF.

This study is in line with the hypothesis that the action value calculations underpin adaptive modulation of social behaviour for relevant social contexts in infants. It has been suggested that animals calculate action values through the integration of external sensory signals with current internal states, and these decisions ideally lead to optimal behavioural choices even in a situation requiring an immediate response, such as a situation of encountering a predator [46]. In humans, it has been reported that stimuli and actions that are uniquely encountered in social interactions can reinforce behaviour through neural mechanisms similar to those underlying non-social reinforcement with money, suggesting that humans calculate the expected social rewards in social interactions [47]. Social interaction and communication provide crucial opportunities for human infants to learn about the external environment. For example, following another's gaze direction plays an important role in language learning [48,49] and detecting potential threat sources [50]. Thus, infants' GF behaviour may be optimized for social learning or avoiding potential threats in each social context.

A remaining question is how GF behaviour is optimized throughout early development. The narrative of GF emergence as described by cognitive theories of GF such as natural pedagogy [3] and perceptual narrowing [51] can account for developing GF behaviour within the first year of life. These theories explain the behavioural bias towards following the gaze in specific situations and how infant GF becomes attuned. However, these theories fail to provide a more general account of contextual modulation of infant GF beyond the specific context modelled in each theory. For example, it has been reported that 20-month-olds show GF equally in situations with and without ostensive cues [52]. Also, 12- to 18-month-olds followed their carers' and strangers’ gaze equally [53]. It is considered that contextual cues become less effective in determining GF after the first year of life. A computational study suggested that infants tend to show GF regardless of contextual factors after they have learnt the action value of GF [54]. Thus, contextual cues might have greater weight in affecting reward expectations and GF in the earlier stage of development, when infants have not yet learnt the action value of GF behaviour. During this early stage, contextual cues such as ostensive cues may help infants disambiguate the context and expect rewards of social interactions, which then facilitate infant GF. Infants could then update the assignment of the action value of GF through the extensive experience of social communication through their development. This perspective could explain the inconsistencies within the literature on infant GF, especially why specific contextual cues modulate GF early in development but then infants start to follow gaze in a wider context in later development, not requiring scaffolding through additional contextual cues.

Future studies should also address what is rewarding for infants in social interactions. On the one hand, Tomasello [55] argued that social engagement such as joint attention is rewarding for infants, due to the evolution of collaborative activities and sharing, and not by the information richness. On the other hand, adults' social signals could be rewarding because they are informative, as it has been shown that informative value can be a reinforcement value. Humans choose options that will provide more information when making decisions [56], and acquiring information enhances brain activations in the reward system, thereby modulating learning performance [57]. Action value for acquiring information can be considered a reinforcement value for humans and it modulates behavioural decision-making. In GF situations, there could be a mixture of two rewards—the social reward of joint attention and the reward of acquiring information about the environment. Future studies could further examine how infants assign a reward value to adults’ social signals in GF situations, and a wider range of interactive social contexts.

Future studies should also explore the generalizability of the mechanisms underlying contextual modulation of GF, especially in more naturalistic or ecologically relevant settings. GF studies in experimental settings have mainly focused on the functional explanation of a particular experimental manipulation of available contextual cues, such as the presence of eye-catching actions [58], the facial familiarity of interacting partners [34], the contingent reactivity [59] and the experience of social interaction before GF tasks [60]. In the experimental settings of GF situations, our study is based on previous studies [7,8,33], and typically, only two objects are presented and GF occurs with a probability of 50% applying the definition of the first fixation to a target object. Conversely, in a naturalistic context, the surrounding environment is more complex with many objects, and infants show a wider repertoire of behaviour than in a screen-based experiment, which greatly reduces the probabilities of infants' GF in each event far below 50% [16,61]. Therefore, we need to be careful not to simply generalize our findings on a screen-based eye-tracking study to a wide range of GF studies, many of which are assessed in more naturalistic and complex contexts. It is necessary to examine how contextual factors modulate infants’ GF in naturalistic situations before any such generalization can be made.

## 5. Conclusion

Here, we showed that the facilitation effects of different social cues (reliability and EC) on infant GF were mediated by increased HR, which may index reward expectations. Infant GF can be hypothesized as being a result of the value-driven decision-making process, which enables infants to actively select when, and from whom, to learn during social communication.

## Data Availability

Data used in the analysis are available in the electronic supplementary material [62].
